# Transformation of the Metastable Al_6_Fe Intermetallic Phase during Homogenization of a Binary Al-Fe Alloy

**DOI:** 10.3390/ma14237208

**Published:** 2021-11-26

**Authors:** Jože Arbeiter, Maja Vončina, Barbara Šetina Batič, Jožef Medved

**Affiliations:** 1 Department for Materials and Metallurgy, Faculty of Natural Sciences and Engineering, University of Ljubljana, Aškerčeva 12, 1000 Ljubljana, Slovenia; maja.voncina@ntf.uni-lj.si (M.V.); jozef.medved@ntf.uni-lj.si (J.M.); 2 Institute of Metals and Technology, Lepi Pot 11, 1000 Ljubljana, Slovenia; barbara.setina@imt.si

**Keywords:** homogenization, aluminum alloys, phase transformation, differential scanning calorimetry

## Abstract

Within the scope of this research the transformation of the Al_6_Fe metastable phase was analyzed via Differential Scanning Calorimetry (DSC), optical and Scanning Electron Microscopy (SEM) and X-ray Diffraction (XRD). A binary Al-Fe1.1 low-impurity alloy was produced with refined raw materials in a controlled environment. With a cooling rate of 35 K/s, solidification of the Al_6_Fe metastable phase was achieved. The samples were homogenized at 600 °C for 2–24 h. Results of a qualitative analysis of metallographic samples show that the transformation began on grain boundaries, forming an Fe-phase free region, but after 2 h began to take place within the eutectic region. The transformation is mostly complete after 12 h, but after 24 h of homogenization it is fully complete as all samples, except the 24 h homogenized one, contain both the metastable Al_6_Fe and the stable Al_13_Fe_4_ phase.

## 1. Introduction

Iron is not only the most common impurity in aluminum alloys but is also a broadly used alloying element for aluminum foil production. Under non-equilibrium solidification conditions at high cooling rates, metastable Al-Fe eutectics may solidify. With proper homogenization annealing, the transformation of metastable eutectics can be achieved, resulting in favorable properties for further thermo-mechanical processing.

Homogenization is an important part of the aluminum alloy production process, as certain irregularities or inhomogeneities that occur during non-equilibrium solidification can be removed with suitable homogenization annealing. Defects in the microstructure of alloys can cause problems in further thermomechanical treatments [1,2,3,4,5,6]. Homogenization annealing enables the elimination of microsegregations and non-equilibrium eutectics, which, if not removed, can cause the material to rupture during further deformation [7,8].

Iron is the most common impurity in aluminum alloys as it can be introduced into alloys from a variety of sources, and at the same time it is very difficult to remove during aluminum recycling. Iron is mostly used as an alloying element in aluminum foil production [9]. Aluminum and iron form the equilibrium Al_13_Fe_4_ phase, but non-equilibrium solidification of alloys containing iron as an alloying element or impurity can lead to the formation of various metastable intermetallic phases, such as Al_6_Fe, Al_m_Fe or Al_x_Fe [10,11,12]. By homogenizing the alloys, the transformation of metastable phases into stable Al_13_Fe_4_ is achieved. The rate of homogenization is determined by the diffusion of alloying elements through the aluminum matrix. The most important parameters influencing the rate of homogenization are time and temperature.

The formation and transformation of the Al_6_Fe metastable intermetallic phase into the stable Al_13_Fe_4_ intermetallic phase have been studied by different authors and methods [10,13,14,15,16,17,18,19,20,21,22,23,24,25,26]. During non-equilibrium solidification of Al-Fe alloys, due to increased cooling rates, many different metastable intermetallic phases can form in addition to the stable Al_13_Fe_4_ phase [25]. The formation of these phases depends on the Al:Fe ratio, the cooling rate, other alloying or trace elements, the amount and composition of the grain refiner, etc. Hollingsworth [14] was the first to discover the metastable intermetallic Al_6_Fe phase containing 25.6 wt % Fe formed in rapidly cooled alloys from the Al-Fe system. The phase was defined by an orthorhombic crystal lattice of the Al_6_Mn type, with a space group CC/mm and lattice parameters a = 0.6492 nm, b = 0.7437 nm and c = 0.8788 nm and a density γ = 3.45 g/cm^3^.

It was also found that at cooling rates between 10^−1^ K/s and 10 K/s, a mixture of Al_13_Fe_4_ and Al_x_Fe intermetallic phases is generally formed, at cooling rates between 2 and 5 K/s a mixture of Al_x_Fe and Al_6_Fe intermetallic phases can form, and at rates above 20 K/s, a metastable Al_m_Fe phase or a metastable Al_9_Fe_2_ phase can be obtained [26]. Researchers later found that at cooling rates up to 0.5 K/s only the Al_13_Fe_4_ phase is formed during solidification, in the range between 0.5 and 0.9 K/s Al_13_Fe_4_ and Al_x_Fe are formed, in the range 0.9–3 K/s only Al_x_Fe is formed, at 3–6 K/s a combination of Al_x_Fe and Al_6_Fe is formed, and above 6 K/s Al_6_Fe is formed. However, the limit above which Al_m_Fe or Al_9_Fe_2_ are formed is unknown. These phases occur at very high cooling rates. It is currently assumed that the formation of so many different metastable phases at higher cooling rates is due to increased supercooling and changes in the nucleation conditions and growth of intermetallic phases. It has also been considered that all the different metastable phases may form due to the influence of other alloying elements [15,16].

The subject of this research was the analysis of the metastable phase transformation during the homogenization of a binary Al-Fe alloy. An extensive study of the transformation of the Al_6_Fe metastable intermetallic phase into the stable Al_13_Fe_4_ intermetallic phase has been studied via Differential Scanning Calorimetry (DSC) and metallographic analysis in order to determine the rate of transformation of the aforementioned phases in a binary low-impurity AlFe1.1 alloy. DSC analysis was used to confirm that the samples contained both the metastable and stable Al-Fe phase and to determine the time necessary to transform the metastable phase into the stable phase at homogenization conditions. With further optical and scanning electron microscopy (SEM) the presence of both phases was confirmed and the results also provided a unique outlook into how the transformation area migrates throughout the microstructure. The X-Ray Diffraction analysis (XRD) confirmed the presence of the Al_6_Fe metastable and the Al_13_Fe_4_ stable intermetallic phase in the analyzed samples.

The main objective of the research was to show at what time at certain homogenization conditions the metastable phase is transformed, which allows more favorable mechanical and microstructural properties for further thermomechanical processing.

## 2. Materials and Methods

Samples were manufactured in a controlled laboratory environment. The aim of the preparation of laboratory-grade alloys was to achieve a microstructure consisting mostly of primary α_Al_ crystals and the Al_6_Fe metastable phase, which cannot be achieved in an industrial environment. The samples were prepared in an induction furnace by combining 99.99 wt % pure refined aluminum and 99.99 wt % pure iron. To produce the alloy without impurities, all instruments, molds and pots were coated with a thin layer of boron nitride (BN). Casting was carried out in a steel mold which was rod-shaped, 160 mm long and had a diameter of 15 mm. In accordance with the literature [12], a cooling rate between 30 and 40 K/s was chosen for the solidification of the metastable Al_6_Fe phase. To achieve the desired cooling rate, the mold was preheated to 450 °C. A type K thermocouple was inserted at the bottom of the mold and the cooling rate of 34.2 K/s was measured during casting. The exact chemical compositions of the alloy produced are listed in Table 1 and the measurements were carried out by means of inductively coupled plasma (ICP) spectroscopy.

**Figure 1:**
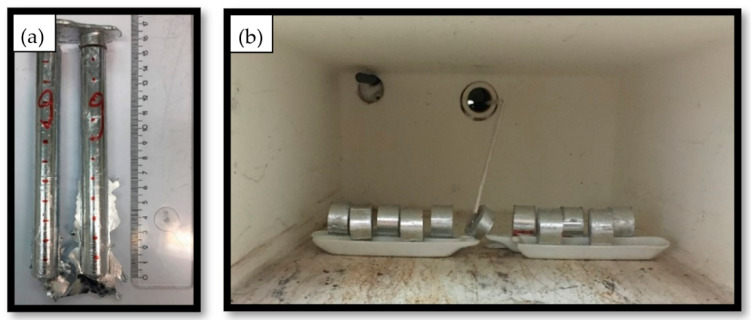
Sample production: (**a**) rod-shaped casting with marked sample sizes, (**b**) sample positioning in the annealing furnace with dummy sample and attached type-K thermocouple.

To perform homogenization annealing, the rods were cut into 10 mm sections using a water-cooled circular saw (Struers, Copenhagen, Denmark). To ensure that all specimens were the same size, the thickness of the cutting disc was considered when cutting. The lower and upper 15 mm parts were removed from the whole rod to ensure a uniform structure of all samples. Homogenization annealing was performed in an electric chamber furnace. Prior to this, the temperature profile of the furnace was measured, and to ensure that the desired temperature was achieved a dummy sample with a type K thermocouple was placed in between the samples to control the internal temperature of the furnace. The cast rods are presented in Figure 1a and the position of the cut samples in the annealing furnace is presented in Figure 1b.

The start of homogenization was recorded when the temperature reached the desired temperature of 600 °C. After 2 h of isothermal annealing, the first sample was removed from the furnace, and after that each subsequent sample was removed at the scheduled time of homogenization. Each sample was immediately quenched in water. Samples were homogenized for 2 h, 4 h, 6 h, 12 h and 24 h. All samples were labelled with the alloy composition and homogenization time in hours. The first sample, which was analyzed in the as-cast state, was labelled as sample AlFe1.1-0, whereas all homogenized samples were labelled as AlFe1.1-2, AlFe1.1-4, AlFe1.1-12, AlFe1.1-18 and AlFe1.1-24.

**Figure 2:**
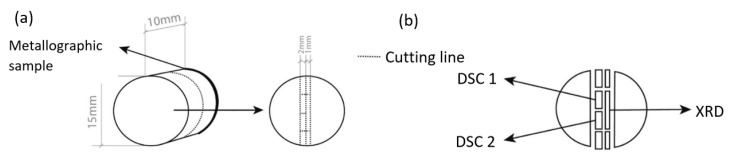
Scheme of sample preparation: (**a**) Preparation of metallographic sample, (**b**) preparation of DSC and XRD samples.

After homogenization, the samples were cut into smaller pieces that were used for different types of analysis. First, the samples were cut in half along the cross section as shown in Figure 2. One half was used to prepare samples for metallographic analysis and the other half was used to prepare samples for DSC and XRD measurements. A 2.0 mm thick slice was cut from the center of the sample using a water-cooled precision circular saw, from which two identical samples were cut in the center for DSC analysis. The second slice was cut to a thickness of 1.0 mm, and the center of this slice was used for XRD.

Thermodynamic characterization of the samples was carried out using DSC, which allows the definition of characteristic melting temperatures and melting enthalpy of the samples, providing an insight into the development of thermal processes that take place during homogenization. DSC tests were performed using an STA Jupiter 449C from Netzsch (Selb, Germany). All measurements were performed in an inert atmosphere with argon gas, with a heating rate of 10 K/min from room temperature to 750 °C. For microstructural analysis, an optical microscope ZEISS Axio Imager A1m (Zeiss, Oberkochen, Germany) equipped with an AxioCam ICc 3 (3.3 megapixel) digital imaging camera (Zeiss, Oberkochen, Germany) and AxioVision image processing and analysis software (version 4.8) was used. For a more detailed analysis of the microstructural elements, the scanning electron microscope FEG-SEM ThermoFisher Scientific Quattro S (Waltham, Massachusetts, USA) was used. Finally, X-Ray Diffraction analysis (XRD, Malvern Panalytical X’Pert using Cu anode, Malvern, UK) was performed in order to confirm the presence of the Al_6_Fe metastable intermetallic phase and the Al_13_Fe_4_ stable intermetallic phase.

## 3. Results and Discussion

**Figure 3:**
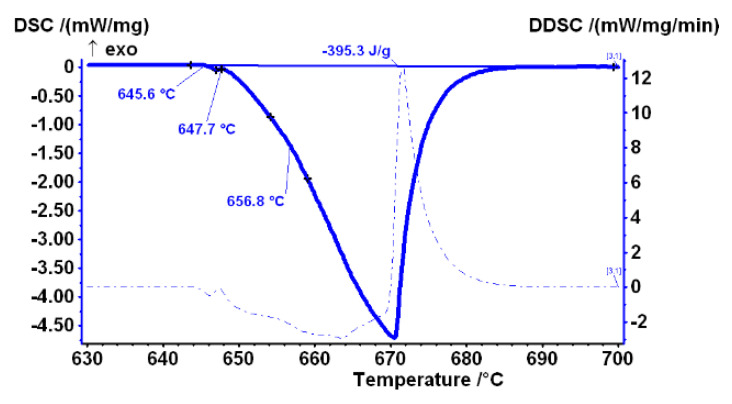
DSC heating curves of sample AlFe1.1-0 in temperature range 630–700 °C, with associated derivative, and marked characteristic temperatures and melting enthalpy.

DSC was performed on all samples. The curve presented in Figure 3 shows the measurement results of the heating curve of sample AlFe1.1-0 in the temperature range 630–700 °C, where the entire melting process of the sample is recorded. According to the literature review [14,15], the initial drop, which can be observed in the curve at 645.6 °C, is associated with the melting of the Al_6_Fe metastable phase. The second characteristic temperature at 647.7 °C indicates the beginning of the melting of the Al_13_Fe_4_ stable phase, and the third at 656.8 °C is the melting of α_Al_.

**Figure 4:**
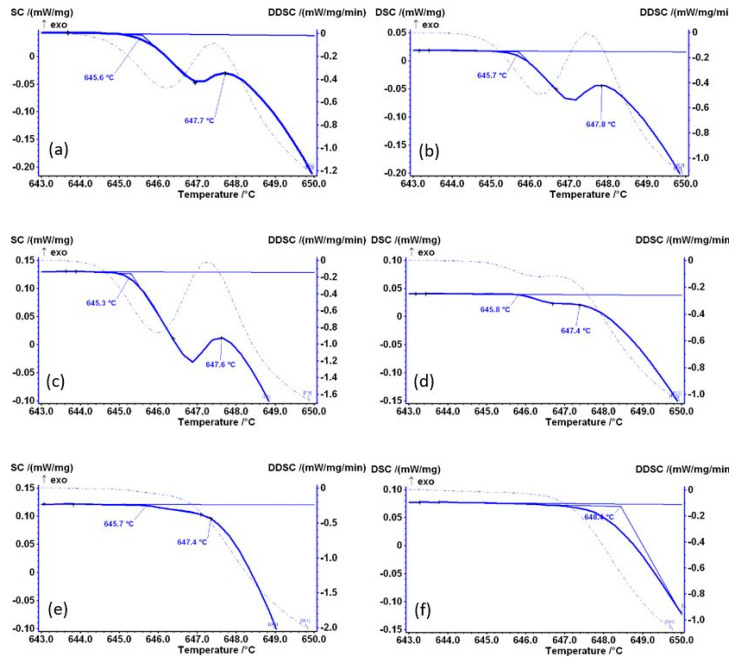
Temperature range 643–650 °C of DSC heating curves with associated derivative and marked characteristic temperatures of samples: (**a**) AlFe1.1-0, (**b**) AlFe1.1-2, (**c**) AlFe1.1-4, (**d**) AlFe1.1-12, (**e**) AlFe1.1-18 and (**f**) AlFe1.1-24.

Figure 4 shows the temperature range 643–650 °C, where the start of melting is recorded. This initial melting of the Al_6_Fe metastable phase is also visible in subsequent diagrams presented in Figure 4, although the intensity of this part of the diagram decreased significantly from sample AlFe1.1-4 onwards (Figure 4c). For samples AlFe1.1-12 (Figure 4d) and AlFe1.1-18 (Figure 4e), this effect is barely visible, indicating that these samples contain a lower amount of the Al_6_Fe metastable phase, whereas the melting of the Al_6_Fe metastable phase is no longer visible in sample AlFe1.1-24, indicating the transformation is complete before 24 h of homogenization at 600 °C.

**Figure 5:**
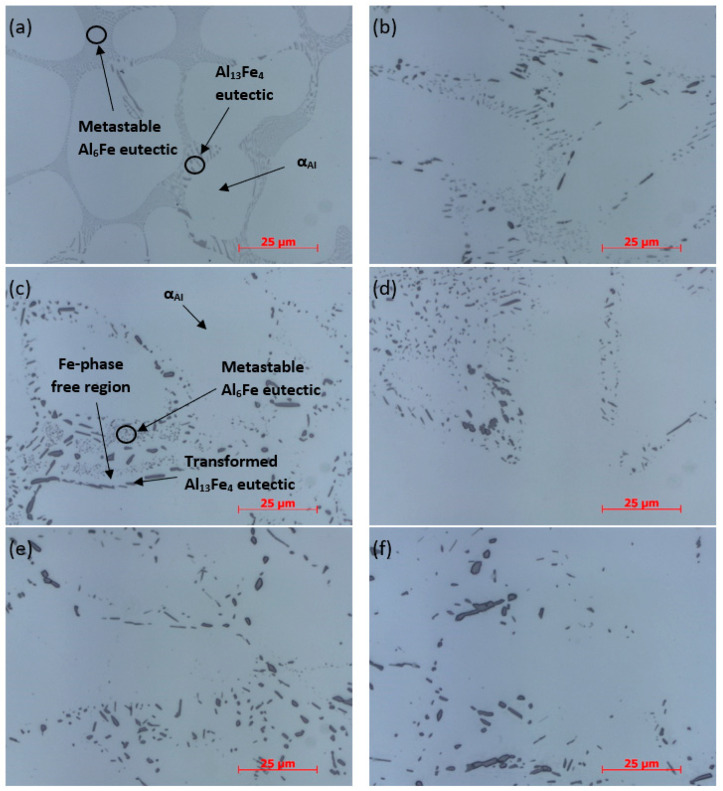
Optical micrographs of the examined aluminum alloy, presenting the microstructures of the as-cast sample and the 2–24 h homogenized samples: (**a**) as-cast state, (**b**) AlFe1.1-2, (**c**) AlFe1.1-4, (**d**) AlFe1.1-12, (**e**) AlFe1.1-18 and (**f**) AlFe1.1-24.

All samples were analyzed by optical microscopy (Figure 5). At 1000× magnification, differences in shape and size of intermetallic phases between the samples can be seen. In sample AlFe1.1-0, very fine spherical particles and rod-shaped phases can be seen, possibly representing the same metastable phase. A slightly darker and sharper phase is also visible, which could represent a stable Al_13_Fe_4_ phase. In the AlFe1.1-2 sample, the conglomeration of the metastable phase particles can be observed, mainly along the boundaries between the dendrite and the eutectic region. As the duration of homogenization increases, the growth of the Al_13_Fe_4_ stable phase in this region becomes noticeable, while the particles of the phase in the eutectic region grow. From the optical microscopy images, it is difficult to predict whether this process is only the growth of particles of the metastable phase or whether the transformation of the metastable phase into the stable phase is taking place. For times over 2 h of homogenization at 600 °C, an Fe-phase free region appears between the stable phase adjacent to the α_Al_ and the metastable phase, suggesting that the elements of the metastable phase have dissolved in this region, which caused stable phase growth at the α_Al_ boundary. Such an effect is particularly noticeable in sample AlFe1.1-4 (Figure 5c).

With increasing homogenization time, the proportion of fine spherical particles of the metastable phase decreases significantly. In sample AlFe1.1-12, such areas are hardly recognizable. It is also noticeable that the transformation of the Al_6_Fe metastable phase migrates from the grain boundary region to the eutectic region with homogenization progression. It is proposed that the diffusion length of Fe atoms becomes too long to diffuse from the dissolving of Al_6_Fe particles to the already existing Al_13_Fe_4_ particles at the grain boundaries, and it is thermodynamically more favorable for the nucleation of new Al_13_Fe_4_ particles within the eutectic region to occur. Thus, the growth of these newly nucleated particles can proceed and the transformation of the Al_6_Fe metastable phase to the Al_13_Fe_4_ stable intermetallic phase can continue.

**Figure 6:**
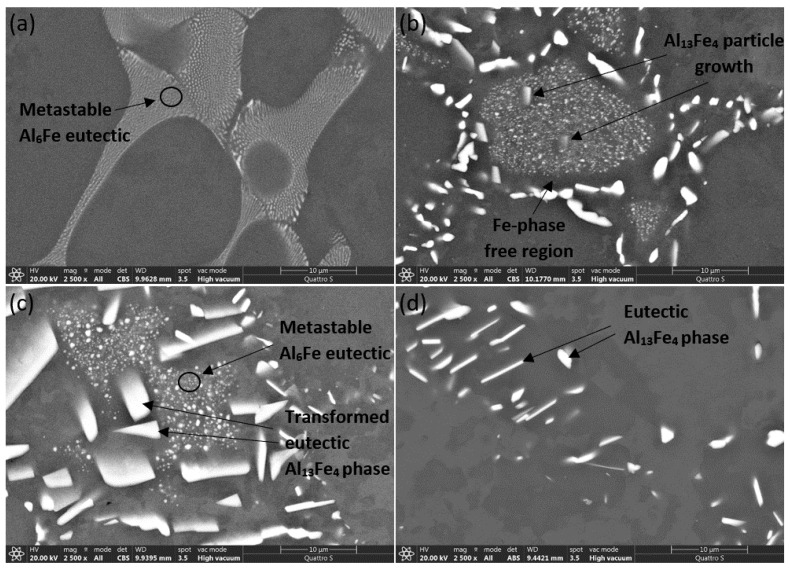
SEM micrographs of samples (**a**) AlFe1.1-0, (**b**) AlFe1.1-4, (**c**) AlFe1.1-12 and (**d**) AlFe1.1-24.

The micrographs shown in Figure 6 were taken with a scanning electron microscope at 2500× magnification. Examination of the micrographs of samples AlFe1.1-0, AlFe1.1-4, AlFe1.1-12 and AlFe1.1-24 confirmed the assumptions of the previous analyses. The predominant phase appearing in the microstructure of sample AlFe1.1-0 is a very fine spherical shape, to which we attribute the Al_6_Fe metastable structure. After homogenization, sample AlFe1.1-4 shows the growth of fine particles in the middle of the eutectic region. It is suspected that this phenomenon is merely the growth of metastable particles, as previous DSC measurements clearly indicate the existence of a metastable phase. The transformation of the metastable phase into a stable phase takes place at the boundary between the eutectic region and the α_Al_ matrix. In this region, the formation and growth of larger particles of the Al_13_Fe_4_ stable phase is observed throughout the sample. The transformation occurs due to the nucleation of Al_13_Fe_4_ particles at the edge of the α_Al_ matrix, which are thermodynamically more stable than metastable particles under the given conditions, whereby the elements of the metastable particles begin to dissolve into the matrix and the excess iron atoms diffuse from the metastable particles to the stable ones. The consequence of this process is the appearance of an Fe-phase free region between the stable and metastable phases, which expands with increasing homogenization time. When combining the results from the optical and scanning electron microscopy, a progression of the transformation can be observed as the particles of the Al_13_Fe_4_ phase grow and the Fe-phase free region expands. Such a phenomenon can be observed in sample AlFe1.1-4, where the growth of the Al_13_Fe_4_ particles are visible. As was determined by analyzing the DSC results, the transformation of the metastable phase is not complete within 12 h of homogenization. Although areas containing the metastable phase were rarely found in the AlFe1.1-12 microstructure, the metastable phase was still present as confirmed by the AlFe1.1-12 SEM micrograph (Figure 6c). The AlFe1.1-24 SEM micrograph (Figure 6d) provides no evidence of the metastable phase, confirming previous results.

**Figure 7:**
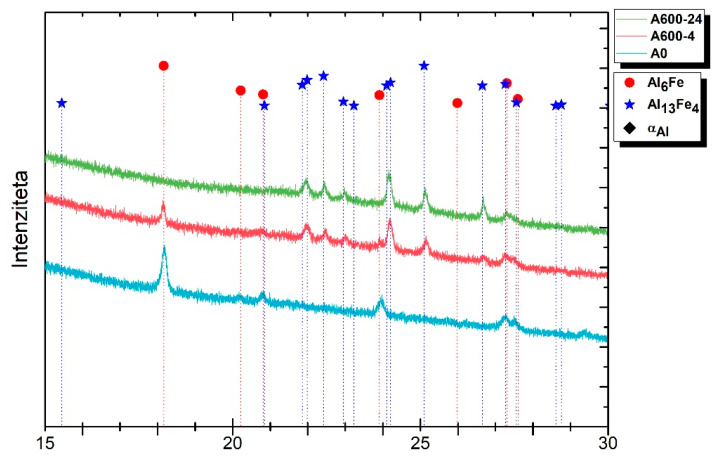
XRD results of samples AlFe1.1-0, AlFe1.1-4 and AlFe1.1-24, confirming the existence of the Al_6_Fe metastable phase in samples AlFe1.1-0 and AlFe1.1-4 and the confirmation of the complete transformation of the Al_6_Fe metastable phase into the stable Al_13_Fe_4_ phase in sample AlFe1.1-24.

XRD was performed on three select samples (Figure 7). Sample AlFe1.1-0 was analyzed in order to confirm the presence of the Al_6_Fe metastable phase because of non-equilibrium solidification, whereas the AlFe1.1-4 and AlFe1.1-24 samples were analyzed to confirm the transformation of the Al_6_Fe metastable phase into the stable Al_13_Fe_4_ intermetallic phase. The peaks at 2Θ 18.166°, 20.815°, 23.908° and 27.306° confirm the presence of the Al_6_Fe metastable phase in sample AlFe1.1-0, but on the other hand, no peaks are visible at these 2Θ angles in the AlFe1.1-24 sample, indicating the completion of the transformation. Peaks at 2Θ 20.984°, 22.422°, 24.205°, 25.100° and 26.652° confirm the presence of the Al_13_Fe_4_ intermetallic phase in sample AlFe1.1-24. The AlFe1.1-4 sample confirms that the transformation is still in progress, due to the fact that both peaks indicating the presence of the Al_6_Fe metastable phase (18.166°) and the Al_13_Fe_4_ stable intermetallic phase (20.984°, 22.422°, 24.205°, 25.100° and 26.652°) are visible.

## 4. Conclusions

Non-equilibrium solidification of binary Al-Fe alloys may result in the formation of metastable eutectics. Results confirm that the cooling rate between 30 and 40 K/s is adequate to produce the Al_6_Fe metastable intermetallic phase in the binary Al-Fe system during casting. It was also confirmed that by homogenizing the AlFe1.1 alloy at 600 °C, after 12 h, most of the Al_6_Fe metastable phase is transformed into the stable Al_13_Fe_4_, but it is only after 24 h that the alloy no longer contain any Al_6_Fe particles and that the transformation of the metastable phase into the stable Al_13_Fe_4_ has been completed. The transformation begins on the boundary between the dendrite and the eutectic area and continues this way for approximately 2 h, after which time the slow diffusion of Fe atoms begins to hinder this progression. This causes the transformation to continue within the eutectic area where Fe atoms have a much shorter diffusion range, even though the nucleation of new Al_13_Fe_4_ stable particles is usually thermodynamically less favorable. The transformation of the metastable phase is dependent on the diffusion rate of Fe atoms within the aluminum matrix.

## Figures and Tables

**Table 1:** Chemical composition of the manufactured aluminum alloy.

Element	Fe	Si	Ti	Cr	Pb	Cu	Mn	Zn	Al
wt %	1.10	<0.01	<0.01	<0.01	<0.01	<0.01	<0.01	<0.01	rest

## Data Availability

The data presented in this study are available on request from the corresponding author.

## References

[1] Mondolfo L.F. (1976). Aluminum Alloys: Structure and Properties.

[2] Davis J.R. (2001). Alloying: Understanding the Basics.

[3] Arbeiter J. (2021). Modelling of Phase Equilibrium and Homogenization Kinetics of Al-Fe and Al-Fe-Si Alloys. Ph.D. Thesis.

[4] ASM International (1990). Properties and Selection: Nonferrous Alloys and Special-Purpose Materials.

[5] Arbeiter J. (2014). Determination of the Optimal Homogenization Temperature of Aluminium Alloys 1XXX and 8XXX Depending on the Amount of Fe and Si. Bachelor’s Thesis.

[6] Vončina M., Kresnik K., Volšak D., Medved J. (2020). Effects of Homogenization Conditions on the Microstructure Evolution of Aluminium Alloy EN AW 8006. Metals.

[7] Hatch J.E. (1984). Aluminum Properties and Physical Metallurgy.

[8] Davis J.R. (1984). Aluminum and Aluminum Alloys ASM Specialty Handbook.

[9] Santora E., Berneder J., Simetsberger F., Doberer M., Chesonis C. (2019). Mechanical Properties Evolution for 8xxx Foil Stock Materials by Alloy Optimization—Literature Review and Experimental Research. Light Metals.

[10] Vončina M., Nagode A., Medved J., Paulin I., Žužek B., Balaško T. (2021). Homogenisation Efficiency Assessed with Microstructure Analysis and Hardness Measurements in the EN AW 2011 Aluminium Alloy. Metals.

[11] Belov N.A., Aksenov A.A., Eskin D.G. (2002). Iron in Aluminum Alloys: Impurity and Alloying Element.

[12] Belov N.A., Eskin D.G., Aksenov A.A. (2005). Multicomponent Phase Diagrams, Applications for Commercial Aluminum Alloys.

[13] Chen J., Dahlborg U., Bao C.M., Calvo-Dahlborg M., Henein H. (2011). Microstructure Evolution of Atomized Al-0.61 wt pct Fe and Al-1.90 wt pct Fe Alloys. Metall. Mater. Trans. B.

[14] Hollingsworth E.H., Willett R.E., Frank G.R. (1962). Identification of a new Al-Fe constituent, FeAl_6_. Tran. Metall. AIME.

[15] Allen C.M., O’Reilly K.A.Q., Evans P.V., Cantor D.B. (1997). A Calorimetric Evaluation of the Role of Impurities in the Nucleation of Secondary Phases in 1xxx Al Alloys. MRS Online Proc. Libr..

[16] Allen C.M., O’Reilly K.A.Q., Cantor B., Evans P.V. (1998). Intermetallic phase selection in 1XXX Al alloys. Prog. Mater. Sci..

[17] Liang D., Korgul P., Jones H. (1996). Composition and solidification microstructure selection in the interdendritic matrix between primary Al_3_Fe dendrites in hypereutectic AlFe alloys. Acta Mater..

[18] Liang D., Jones H. (1992). The effect of growth velocity on growth temperature of the Al-Al_3_Fe and Al-Al_6_Fe eutectics. Z. Metallk..

[19] Walford L.K. (1965). The structure of the intermetallic phase FeAl6. Acta Crystallogr..

[20] Shillington E. (1996). Thermal Stability of Metastable Intermetallic Phases in Model Al-Fe and Al-Fe-Mn-Si Alloys, Part II. Ph.D. Thesis.

[21] Lendvai J., Rajkovitz Z., Ungar T., Kovads I. (1986). Formation and reversion of precipitates in a DC cast Al-0.5% Fe alloy. Aluminium.

[22] Tonejc A. (1971). X-ray study of the decomposition of metastable Al-rich Al-Fe solid solutions. Metall. Mater. Trans. B.

[23] Kosuge H., Mizukami I. (1972). Behavior of fir-tree structure in Al-Fe-Si alloy ingots soaking at elevated temperatures. J. Jpn. Inst. Light Met..

[24] Jones H. (1978). Developments in Aluminium Alloys by Solidification at Higher Cooling rates. Aluminum.

[25] Totten G.E., Mackenzie D.S. (2003). Handbook of Aluminum: Volume 1: Physical Metallurgy and Processes.

[26] Young R.M.K., Clyne T.W. (1981). An A1-Fe Intermetallic Phase Formed During Controlled Solidification. Scr. Metall..

